# Lactic acid bacteria affect serum cholesterol levels, harmful fecal enzyme activity, and fecal water content

**DOI:** 10.1186/1476-511X-8-21

**Published:** 2009-06-11

**Authors:** Do Kyung Lee, Seok Jang, Eun Hye Baek, Mi Jin Kim, Kyung Soon Lee, Hea Soon Shin, Myung Jun Chung, Jin Eung Kim, Kang Oh Lee, Nam Joo Ha

**Affiliations:** 1 Department of Pharmacy, Sahmyook University, Seoul 139-742, Republic of Korea; 2 Department of Nursing, Sahmyook University, Seoul 139-742, Republic of Korea; 3 College of Pharmacy, Duksung Women's University, Seoul 132-714, Republic of Korea; 4 Cellbiotech Co Ltd, Seoul 157-030, Republic of Korea; 5 Department of Life Science, Sahmyook University, Seoul 139-742, Republic of Korea

## Abstract

**Background:**

Lactic acid bacteria (LAB) are beneficial probiotic organisms that contribute to improved nutrition, microbial balance, and immuno-enhancement of the intestinal tract, as well as lower cholesterol. Although present in many foods, most trials have been in spreads or dairy products. Here we tested whether *Bifidobacteria *isolates could lower cholesterol, inhibit harmful enzyme activities, and control fecal water content.

**Methods:**

*In vitro *culture experiments were performed to evaluate the ability of *Bifidobacterium *spp. isolated from healthy Koreans (20~30 years old) to reduce cholesterol-levels in MRS broth containing polyoxyethanylcholesterol sebacate. Animal experiments were performed to investigate the effects on lowering cholesterol, inhibiting harmful enzyme activities, and controlling fecal water content. For animal studies, 0.2 ml of the selected strain cultures (10^8^~10^9 ^CFU/ml) were orally administered to SD rats (fed a high-cholesterol diet) every day for 2 weeks.

**Results:**

*B. longum *SPM1207 reduced serum total cholesterol and LDL levels significantly (*p *< 0.05), and slightly increased serum HDL. *B. longum *SPM1207 also increased fecal LAB levels and fecal water content, and reduced body weight and harmful intestinal enzyme activities.

**Conclusion:**

Daily consumption of *B. longum *SPM1207 can help in managing mild to moderate hypercholesterolemia, with potential to improve human health by helping to prevent colon cancer and constipation.

## Background

Probiotic bacteria have multiple potential health effects, including blocking gastroenteric pathogens [1-4], neutralizing food mutagens produced in the colon [1,5-10], enhancing the immune response [6,9,11-14], lowering serum cholesterol, and stopping intestinal dysfunction [15-21]. In general, probiotic bacteria must colonize the gastrointestinal tract (GIT) of the host, have acid- and bile salt-tolerance, and block putrefactive bacteria in the GIT. Lactic acid bacteria (LAB), especially *Lactobacillus *spp. and *Bifidobacterium *spp. are important GIT residents and are used as probiotic strains to improve health [22-24]. *Lactobacillus *and *Bifidobacterium *have been used in fermented foods for several centuries without adverse effects [25,26] and are classified as Generally Recognized as Safe (GRAS) because of their long history of safe use, particularly in dairy foods [27,28].

Here, we evaluated the ability of *Bifidobacteria spp*. isolated from healthy Koreans (20~30 years old) to lower cholesterol, inhibit harmful enzyme activities, and control the fecal water content.

## Materials and methods

### Bacterial strains

The origins of the strains used in this study are shown in Table 1. Isolation of *Bifidobacteria *was performed from fecal samples of healthy Koreans (20~30 years old) collected by BBL's anaerobic sample collection and transport system to maintain anaerobic conditions, and were used within 24 h. Fecal samples were serially diluted 10-fold from 10^-1 ^to 10^-8^, and 100 μl was spread onto selective BL agar containing 5% sheep blood. After 48 h of incubation in anaerobic conditions (90% N_2_, 5% H_2_, 5% CO_2_) (Bactron Anaerobic Chamber, Sheldon Manufacturing Inc., USA) at 37°C, brown or reddish-brown colonies 2~3 mm in diameter were selected for further identification [29].

**Table 1:** List of lactic acid bacteria used in this study

Bacterial strains	Source	Origin
*Bifidobacterium adolescentis *SPM1005	Isolate^a^	Human feces
*Bifidobacterium longum *SPM1207	Isolate	Human feces
*Bifidobacterium adolescentis *SPM1601	Isolate	Human feces
*Bifidobacterium adolescentis *KCTC3352	Commercial^b^	Intestine of adult
*Bifidobacterium longum *KCTC3128	Commercial	Intestine of adult
*Lactobacillus acidophilus *(LH) CBT	Commercial	NA^c^
*Lactobacillus plantarum *KCTC1048	Commercial	NA
*Lactobacillus plantarum *(LP) CBT	Commercial	NA
*Enterococcus faecium *SPM1206	Isolate	Human feces

^a^Isolated from healthy Korean

^b^Purchased from Korean Collection for Type Culture (KCTC)

^c^Not available

A fructose-6-phosphate phosphoketolase (F6PPK) test was performed [30] to ensure that the colonies selected were *Bifidobacteria*. To identify the isolated *Bifidobacterium *spp. at the species level, 16s rRNA sequencing was performed by Bioleaders (Daejeon, Korea).

### *In vitro *cholesterol-lowering test

MRS broth (pH7.0) (Difco, USA) containing 0.05% L-cysteine·HCl·H_2_O (w/v) was prepared and autoclaved at 121°C for 15 min. Soluble cholesterol (polyoxyethanyl-cholesterol sebacate, Sigma, USA) was added to the prepared MRS broth and filtered through a 0.45 μm Millipore filter. The inoculation volume was 15 μl of provisional probiotic bacterial culture (10^8^~10^9 ^CFU/ml) solution per 1 ml cholesterol-MRS broth, and that was anaerobically incubated at 37°C for 24 h. Uninoculated MRS broth was also incubated at 37°C for 24 h for the control.

Following incubation, bacterial cells were removed by centrifugation (3,000 rpm, 10 min), and the spent broth and uninoculated control broth were then assayed for their cholesterol content. The remaining volume of cholesterol in the cholesterol-MRS broth was determined by the method reported by Rudel and Morris with a small modification [31]. To measure the amount of cholesterol, the dye layer is observed at 560 nm.

### Experimental animals and diets

A total of 24 Sprague-Dawley (SD) male rats (5-week-old) were purchased from Central Lab Animal Inc. (Korea), and were housed in a temperature-controlled animal room (22 ± 2°C) with a 12 h light/dark cycle and humidity 55 ± 5%. Food and water were freely supplied. The animals were randomly selected and assigned to three groups (8 rats per group) according to the type of diet. Group 1 was fed a normal diet. Group 2 was fed a high-cholesterol diet and saline (as control). Group 3 was fed a high-cholesterol diet and *B. longum *SPM1207 (the best strain at lowering cholesterol *in vitro*). The composition of high-cholesterol feed is shown in Table 2. All the rats were acclimatized to the respective diets for a week before the experiment started. Rats in groups 2 or 3 received daily administrations of 0.2 ml of saline or *B. longum *SPM1207 (10^8^~10^9 ^CFU/ml), respectively, for 2 weeks. Body weight was monitored weekly and food consumption was monitored daily.

**Table 2:** Compositions of high-cholesterol diets for SD rats

Ingredients	Compositions	
	
	g	kcal
Casein (from milk)	200	800
Corn Starch	155.036	620
Sucrose	50	200
Dextrose	132	528
Cellulose	50	0
Soybean Oil	25	225
Lard	175	1575
Mineral Mixture	35	0
Vitamin Mixture	10	40
TBHQ	0.014	0
DL-Methionine	0	0
L-Cystine	3	12
Choline Bitartrate	2.5	0
Total	838	4,000

Cholesterol	20 g/kg	(2%)
Cholic acid	5 g/kg	(0.5%)

### Analysis of blood serum

At the end of the experimental period of 3 weeks, blood samples from each animal were collected into tubes by cardiac puncture to determine the serum cholesterol level. Serum was separated from the blood by centrifugation at 3,500 rpm for 10 min. The total cholesterol, HDL-cholesterol, and LDL-cholesterol were analyzed by Samkwang Lab (Korea).

### Fecal sampling and bacteriological analysis

Fecal samples were collected weekly to determine the number of LAB, harmful enzyme activity, and water content. Fecal samples were taken directly from the rectum by rectal stimulation and immediately transferred into sterile tubes and kept at 4°C. Total LAB counts was performed on MRS-agar and incubated at 37°C for 48 h under anaerobic conditions (90% N_2_, 5% H_2_, 5% CO_2_). The numbers of colony forming units (CFU) are expressed as log_10 _CFU per gram.

### Harmful enzyme activity of rat intestinal microflora

Enzyme activities related to colon cancer were tested in fecal samples of rats as previously described [32-34].

### Tryptophanase activity assay

Tryptophanase activity was assayed using 2.5 ml of a reaction mixture consisting of 0.2 ml of complete reagent solution (2.75 mg pyridoxal phosphate, 19.6 mg disodium EDTA dihydrate, and 10 mg bovine serum albumin in 100 ml of 0.05 M potassium phosphate buffer, pH 7.5), 0.2 ml of 20 mM tryptophan, and 0.1 ml of the enzyme solution (suspended fecal sample), incubated for 1 h at 37°C, and then stopped by adding 2 ml of color reagent solution (14.7 g p-dimethylaminobenzaldehyde in 52 ml H_2_SO_4 _and 948 ml 95% ethanol). The stopped reaction mixture was centrifuged at 3,000 rpm for 10 min and enzyme activity was measured by monitoring absorbance at 550 nm.

### Urease activity assay

Urease activity was assayed using 0.5 ml of a reaction mixture consisting of 0.3 ml of urea substrate solution (4 mM urea in 20 mM sodium phosphate buffer, pH 7.0) and 0.1 ml of the enzyme solution (suspended fecal sample) incubated for 30 min at 37°C and then stopped by adding 0.1 ml of 1 N (NH_4_)_2_SO_4_. Phenolnitroprusside reagent (1 ml) and alkaline hypochlorite reagent (NaClO, 1 ml) were added to the stopped reaction mixture and incubated for 20 min at 65°C. The reaction mixture was centrifuged at 3,000 rpm for 10 min. Enzyme activity was measured by monitoring absorbance at 630 nm.

### β-glucosidase activity assay

β-glucosidase activity was assayed using 2 ml of a reaction mixture consisting of 0.8 ml of 2 mM p-nitrophenyl-β-D-glucopyranoside and 0.2 ml of the enzyme solution (suspended fecal sample), incubated for 30 min at 37°C, and then stopped by adding 1 ml of 0.5 N NaOH. The stopped reaction mixture was centrifuged at 3,000 rpm for 10 min. Enzyme activity was measured by monitoring absorbance at 405 nm.

### β-glucuronidase activity assay

β-glucuronidase activity was assayed using 2 ml of a reaction mixture consisting of 0.8 ml of 2 mM p-nitrophenyl-β-D-glucuronide and 0.2 ml of the enzyme solution (suspended fecal sample), incubated for 30 min at 37°C, and then stopped by adding 1 ml of 0.5 N NaOH. The stopped reaction mixture was centrifuged at 3,000 rpm for 10 min. Enzyme activity was measured by monitoring absorbance at 405 nm.

### Measurement of fecal water content

The water content of fecal samples was measured using a drying oven (105°C, 24 h). Fecal water content (%) is calculated by:



where W_wet _and W_dry _are the weight of the fecal sample before and after drying in the oven.

### Statistical Analysis

Results were expressed as mean ± standard deviation (SD). For statistical evaluation of data, one-way ANOVA was applied using SPSS 13.0 for Windows followed by post hoc comparisons using the Tukey's test. Differences were considered significant at *p *< 0.05.

## Results

### Isolation and characterization of *B. longum *SPM1207

**Figure 1:**
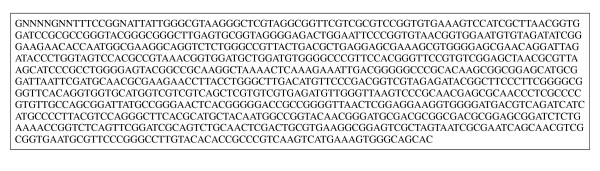
**Sequence analyses of the *B. longum *SPM1207-16s rRNA gene shows 99% identity with *B. longum *DJO10A (879 bp)**.

*B. longum *SPM1207 isolated from healthy Korean faeces was Gram-positive rods, with a translucent glossy colony on general anaerobic medium (GAM, Nissui Pharm. Co. Ltd., Japan) under anaerobic conditions (90% N_2_, 5% H_2_, 5% CO_2_). Sequence analysis (Figure 1) and BLAST searches indicated that the 16s rRNA sequences in this strain showed 99% homology with *Bifidobacterium longum *DJO10A.

### *In vitro *cholesterol-lowering test

Among the tested strains, *B. longum *SPM1207 had the highest cholesterol-reducing activities in MRS broth containing cholesterol (Table 3). On average, *Bifidobacterium *showed higher cholesterol-reducing activities than *Lactobacillus*. And the strains presented different cholesterol lowering effects despite being the same species. The effect of *B. longum *SPM1207 was 2 times higher than *B. longum *KCTC3128.

**Table 3:** Amount of residual cholesterol after *in vitro *incubation of selected lactic acid bacteria

Species	Residual cholesterol (mg/dl)	Cholesterol lowering ratio (%)	*p *value
Control^a^	345.0 ± 5.7^b^	-	-
*Bifidobacterium adolescentis *SPM1005	318.7 ± 3.0	7.6	*p *< 0.05^c^
*Bifidobacterium longum *SPM1207	262.8 ± 1.3	23.8	*p *< 0.05
*Bifidobacterium adolescentis *SPM1601	283.3 ± 2.6	17.9	*p *< 0.05
*Bifidobacterium adolescentis *KCTC3352	329.0 ± 5.6	4.6	*p *< 0.05
*Bifidobacterium longum *KCTC3128	303.8 ± 6.0	11.9	*p *< 0.05
*Lactobacillus acidophilus *(LH) CBT	321.7 ± 1.9	6.7	*p *< 0.05
*Lactobacillus plantarum *KCTC1048	314.0 ± 4.4	9.0	*p *< 0.05
*Lactobacillus plantarum *(LP) CBT	296.3 ± 5.0	14.1	*p *< 0.05
*Enterococcus faecium *SPM1206	300.9 ± 1.7	12.8	*p *< 0.05

^a^MRS broth containing cholesterol without lactic acid bacteria

^b^Each value provided is the mean ± SD

^c^*p *< 0.05 significantly different compared with control

### Lowering of serum cholesterol in rats

We then tested the hypocholesterolemic effects of this LAB in rats fed a high-cholesterol diet. A high cholesterol diet increased serum cholesterol levels (Table 4). *B. longum *SPM1207 treatment reduced total cholesterol from 111.3 to 84.4 mg/dl and LDL-cholesterol levels from 33.3 to 23.5 mg/dl. In addition, *B. longum *SPM1207 slightly increased HDL-cholesterol levels, but did not significantly (Table 4).

**Table 4:** Effects of the *B. longum *SPM1207 on serum total-, HDL-, and LDL-cholesterol of SD rats fed on a high-cholesterol diet

Index	Basal diet	High-cholesterol diet		*p *value
			
		Control	SPM1207^b^	
Total cholesterol (mg/dl)	55.0 ± 5.5^a^	111.3 ± 25.2	84.8 ± 4.3	*p *< 0.05^c^
HDL^e^-cholesterol (mg/dl)	23.9 ± 1.4	30.0 ± 2.6	30.6 ± 1.5	NS^d^
LDL^f^-cholesterol (mg/dl)	11.6 ± 1.1	33.3 ± 8.5	23.5 ± 2.3	*p *< 0.05

^a^Each value provided is the mean ± SD for 8 rats.

^b^*Bifidobacterium longum *SPM1207 isolated from healthy Korean.

^c^*p *< 0.05 significantly different compared with control.

^d^NS: not significant.

^e^HDL: high density lipoprotein,

^f^LDL: low density lipoprotein

### Fecal water content, body weight, and bacteriological analysis

**Figure 2:**
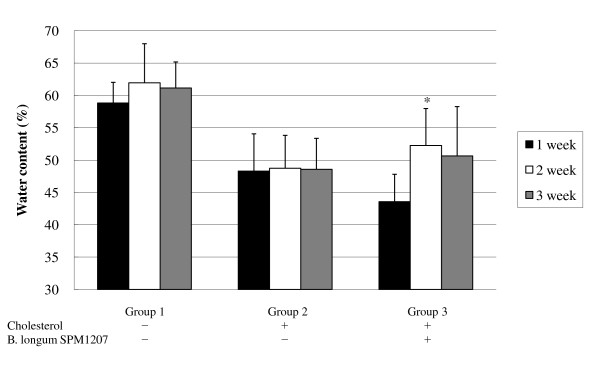
**Effect of *B. longum *SPM1207 on fecal water content**. All rats were acclimatized to the respective diets for a week. Then the rats in group 2 or 3 received daily 0.2 ml saline or *B. longum *SPM1207 (10^8^~10^9 ^CFU/ml), respectively, for 2 weeks. Data are presented as means and standard deviation. *p < 0.05 significantly different compared with 1 week.

**Figure 3:**
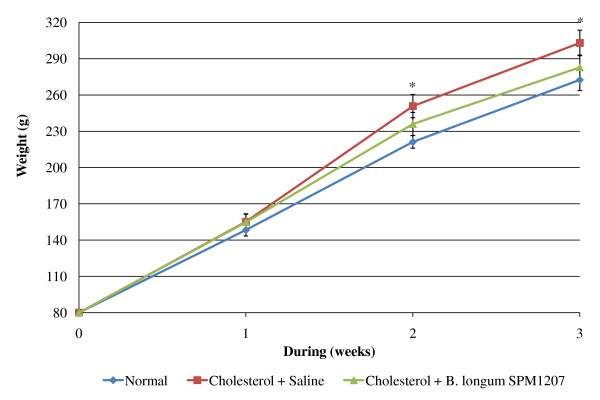
**Changes in body weight fed experimental diets for 3 weeks**. All rats were acclimatized to the respective diets for a week. Then the rats in group 2 or 3 received daily 0.2 ml saline or *B. longum *SPM1207 (10^8^~10^9 ^CFU/ml), respectively, for 2 weeks. Data are presented as means and standard deviation. *p < 0.05 significantly different compared with control (Cholesterol + Saline).

**Figure 4:**
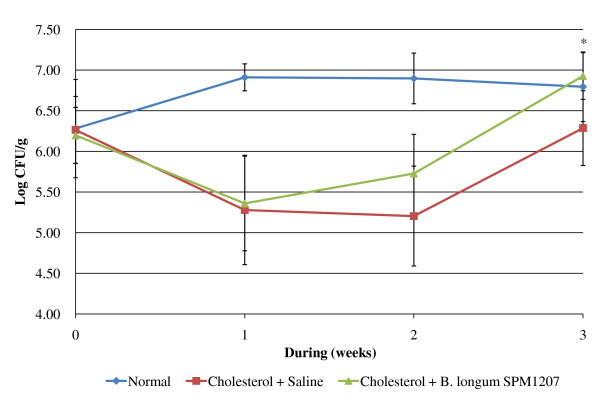
**Changes in total LAB counts in rats fed experimental diets for 3 weeks**. All rats were acclimatized to the respective diets for a week. Then the rats in group 2 or 3 received daily 0.2 ml saline or *B. longum *SPM1207 (10^8^~10^9 ^CFU/ml), respectively, for 2 weeks. Data are presented as means and standard deviation. *p < 0.05 significantly different compared with control (Cholesterol + Saline).

The high cholesterol diet caused dry feces, but *B. longum *SPM1207 treatment increased fecal water content (Figure 2). The high cholesterol diet also increased body weight after 2 weeks, but *B. longum *SPM1207 blocked this increase (Figure 3). Fecal LAB counts were similar in all groups before the experimental diets, but a high-cholesterol diet lowered LAB counts. LAB administration increased fecal LAB counts from 5.3 log_10 _CFU/g to 6.9 log_10 _CFU/g, which was significantly higher than controls (Figure 4).

### Inhibitory effect on harmful enzyme of rat intestinal microflora

**Figure 5:**
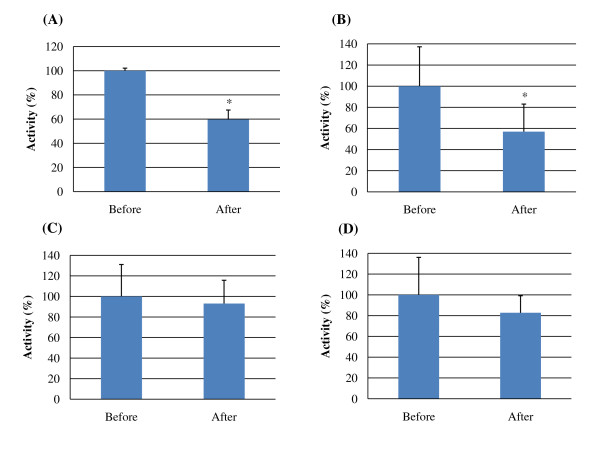
***In vivo *inhibitory effects of *B. longum *SPM1207 on fecal harmful enzymes in rats fed on high-cholesterol diet**. (A) Tryptophanase activity, (B) Urease activity, (C) β-glucosidase activity, (D) β-glucuronidase activity; Before: before the experiment started, After: at the end of experiment.

*B. longum *SPM1207 significantly inhibited tryptophanase and urease activities and slightly decreased β-glucosidase and β-glucuronidase activities (Figure 5).

## Discussion

Cardiovascular disease is the most important cause of death in westernized countries, including Korea. In the United States, 10 million people suffer from ischemic coronary arterial diseases, and spend 115 billion dollars per year to treat it [19]. According to NHANES (the third national health and nation examination survey) data and NCEP (national cholesterol education program) guide, a half million people have died of ischemic cardiac disease. [19,35].

Hypercholesterolemia is strongly associated with coronary heart disease and arteriosclerosis [35-38], and decreasing serum cholesterol is an important treatment option. HDL-cholesterol can prevent arteriosclerosis by removing cholesterol from the blood stream, whereas LDL-cholesterol causes accumulation of cholesterol in blood vessels [35,39]. According to Frick et al. [40], every 1% reduction in body cholesterol content lowers the risk for cardiovascular diseases by 2%. Therapeutic lifestyle changes including dietary interventions, in particular a reduction of saturated fat and cholesterol, are established as a first line therapy to reduce LDL-cholesterol. A change in dietary habits, such as eating fermented products containing lactic acid bacteria, can reduce cholesterol. Since the early studies of Mann and Spoerry [41], the cholesterol-lowering potential of lactic acid bacteria such as *Lactobacillus and Bifidobacterium *is commonly studied *in vitro *or *in vivo *(experimental animals and human subjects) [21,23,36,42-46].

Here, *B. longum *SPM1207 isolated from healthy Korean feces had hypocholesterolemic effects *in vitro *and in experimental animals (from 345.0 mg/dl to 262.8 mg/dl, and 111.3 mg/dl to 84.8 mg/dl, respectively). *B. longum *SPM1207 also slightly increased HDL-cholesterol levels, in agreement with other findings that decreased total cholesterol was accompanied by simultaneous increases of HDL-cholesterol [47,48].

Cholesterol reduction by lactic acid bacteria can be explained by five mechanisms [49-54]: (a) fermentation products of lactic acid bacteria inhibit cholesterol synthesis enzymes and thus reduce cholesterol production; (b) the bacteria facilitate the elimination of cholesterol in feces; (c) the bacteria inhibit the absorption of cholesterol back into the body by binding with cholesterol; (d) the bacteria interfere with the recycling of bile salt (a metabolic product of cholesterol) and facilitate its elimination, which raises the demand for bile salt made from cholesterol and thus results in body cholesterol consumption; and, (e) due to the assimilation of lactic acid.

Lactic acid bacteria have anti-tumor effects [6,8,9,55,56] and block harmful intestinal enzyme activities, a recognized risk factor for colon cancer [8,57,58]. Consumption of *L. rhamnosus *GG decreased the activity of β-glucuronidase [59,60], nitroreductase [60], and cholylglycine hydrolase [60,61]. Consumption of milk enriched with *L. casei *for 4 weeks temporarily decreased β-glucuronidase activity in 10 healthy men but not in 10 healthy control subjects [62]. Consumption of milk fermented with a *Bifidobacterium *species decreased β-glucuronidase activity compared with baseline but did not affect fecal pH or the activity of nitrate reductase, nitroreductase, and azoreductase [63]. Consumption of fermented milk with *L. acidophilus*, *B. bifidum*, *Streptococcus lactis*, and *Streptococcus cremoris *for 3 weeks decreased nitroreductase activity but not β-glucuronidase and azoreductase [64].

Here, *B. longum *SPM1207 decreased tryptophanase, urease, β-glucosidase, and β-glucuronidase in rats. Fecal LAB counts in the *B. longum *SPM1207 feeding group was about 10 times greater than that in the control group, indicating bacterial survival through the gastrointestinal tract.

## Conclusion

In conclusion, the incorporation of *B. longum *SPM1207 in the diet suppressed serum cholesterol levels on a cholesterol-enriched diet. This LAB also improved the balance of the intestinal flora, improved tryptophanase, urease, β-glucosidase, and β-glucuronidase, and increased fecal LAB levels and fecal water content. Therefore, *B. longum *SPM1207 may be a functional probiotic to treat hypercholesterolemia, help prevent colon cancer, and constipation. Studies in humans, however, could be resulted in contradictory outcomes. So, further clinical trials to confirm these effects must be conducted.

## Competing interests

The authors declare that they have no competing interests.

## Authors' contributions

This study was conceived by NJH and designed by NJH, KOL, KSL, and HSS. NJH, KOL, MJC, and JEK were responsible for obtaining funding and sample collection. The in vitro cholesterol-lowering test and animal experiments were done by DKL, SJ, EHB, and MJK. DKL performed data analysis and wrote the draft of the manuscript. All authors read and approved the final manuscript.
